# PD-1 and PD-L1 expression in rare lung tumors

**DOI:** 10.3389/pore.2023.1611164

**Published:** 2023-05-18

**Authors:** Marton Gyulai, Zsolt Megyesfalvi, Lilla Reiniger, Tunde Harko, Bence Ferencz, Luca Karsko, Laszlo Agocs, Janos Fillinger, Balazs Dome, Zoltan Szallasi, Judit Moldvay

**Affiliations:** ^1^ County Institute of Pulmonology, Torokbalint, Hungary; ^2^ Károly Rácz Doctoral School of Clinical Medicine, Semmelweis University, Budapest, Hungary; ^3^ National Koranyi Institute of Pulmonology, Budapest, Hungary; ^4^ Department of Thoracic Surgery, National Institute of Oncology, Semmelweis University, Budapest, Hungary; ^5^ Department of Thoracic Surgery, Comprehensive Cancer Center, Medical University of Vienna, Vienna, Austria; ^6^ Institute of Pathology and Experimental Cancer Research, Semmelweis University, Budapest, Hungary; ^7^ Department of Bioinformatics, Semmelweis University, Budapest, Hungary; ^8^ Computational Health Informatics Program, Boston Children’s Hospital and Harvard Medical School, Boston, MA, United States

**Keywords:** adenoid cystic carcinoma, mucoepidermoid carcinoma, programmed cell death ligand-1 (PD-L1), programmed cell death-1 (PD-1), rare lung tumors

## Abstract

**Background:** Our knowledge is still limited about the characteristics and treatment of rare lung tumors. The aim of our study was to determine programmed cell death ligand-1 (PD-L1) and programmed cell death-1 (PD-1) expression in rare pulmonary tumors to assess the potential role of immunotherapy.

**Methods:** 66 pathologically confirmed rare lung tumors including 26 mucoepidermoid carcinomas (MECs), 27 adenoid cystic carcinomas (ACCs), and 13 tracheobronchial papillomas (TBPs) were collected retrospectively. Immunohistochemical (IHC) staining was performed on formalin fixed paraffin embedded (FFPE) tumor tissues, and PD-L1 expression on tumor cells (TCs) and immune cells (ICs), and PD-1 expression on ICs were determined. The cut off value for positive immunostaining was set at 1% for all markers.

**Results:** PD-L1 expression on TCs was observed in two cases of MEC (7.7%), one case of ACC (3.7%), and was absent in TBP samples. PD-L1 expression on ICs could be demonstrated in nine cases of MEC (34.6%), four cases of ACC (14.8%), and was absent in TBPs. All PD-L1 TC positive tumors were also PD-L1 IC positive. Higher expression level than 5% of PD-L1 TC and/or IC was observed only in one ACC and in two MEC patients. Among them, strong PD-L1 immunopositivity of >50% on TCs and of >10% on ICs could be demonstrated in one MEC sample. PD-L1 expression of ≥1% on ICs was significantly more common in MEC, than in TBP (*p* < 0.001). In MEC ≥1% PD-L1 TC or IC expressions were significantly more common in patients aged 55 or older, than in younger patients (*p* = 0.046, and *p* = 0.01, respectively). PD-1 expression on ICs was found in five cases of MEC (19.2%), four cases of ACC (14.8%), and in two cases of TBP (15.4%). Only one MEC case showed a higher than 5% expression level of PD-1 on ICs.

**Conclusion:** This retrospective study comprehensively demonstrated the rare expression of PD-L1 and PD-1 in pulmonary MEC, ACC, and TBP. However, we found very strong PD-L1 immunopositivity on both TCs and ICs in one MEC sample, which warrants further investigations in a larger cohort.

## Introduction

Lung cancer is the leading cause of cancer death worldwide with a still very low 5-year survival rate of around 20% [1]. During the last decade immunotherapy has revolutionized lung cancer management especially in non-small cell lung cancer (NSCLC). By applying anti PD-1 (programmed cell death-1) and PD-L1 (programmed cell death ligand-1) immune checkpoint inhibitor treatment, unprecedented long survival could be achieved in both adenocarcinoma (ADC) and squamous cell carcinoma (SCC) subtypes of NSCLC [2, 3]. Patient selection for immunotherapy still represents challenges. Although PD-L1 immunohistochemistry (IHC) is not a perfect marker to predict the efficacy of immunotherapy, it is still the most widely used predictive marker in clinical practice [4]. PD-L1 status can be assessed with various primary antibodies and methods with different sensitivity, which limits the comparability of results from different investigations [5].

Rare lung cancer is a very heterogenous group of malignant tumors, which includes adenosquamous lung cancer, pulmonary sarcomatoid carcinoma, primary salivary gland-type lung tumor, and NUT (nuclear protein in testis) carcinoma. Mucoepidermoid carcinoma (MEC) and adenoid cystic carcinoma (ACC) derive from the salivary glands of the tracheobronchial tree and represent 0.1%–0.2% of all lung cancer cases [6]. These tumors are often located endobronchially, therefore, bronchoscopic excision can provide sufficient tissue sample for pathologic diagnosis. In well-localized, early-stage cases mechanical tumor extraction with or without LASER treatment using rigid bronchoscope under general anesthesia is an important part of patient management. In metastatic cases, therapeutic options are often limited and the combination of radiotherapy and chemotherapy yields only modest survival gains [7].

Tracheobronchial papilloma (TBP) is a rare benign lung tumor and in small size cases complete bronchoscopic removal may be a therapeutic solution. However, it has a high recurrence rate, and multiple recurrences may lead to malignant transformation into SCC.

The optimal treatment for MEC and ACC has not yet been determined, especially in metastatic cases. Radiotherapy and chemotherapy are used as systemic treatments, albeit with a very low cure rate [8]. The role of immunotherapy in the management of MEC and ACC is not clear, and PD-L1 expression levels have only been assessed using immunohistochemistry (IHC) in a few ACC cases and have not yet been investigated in MEC and TBP [9].

The aim of our present work was to comprehensively determine PD-L1/PD-1 expression in rare pulmonary tumors to study the potential applicability of immunotherapy.

## Patients and methods

### Ethics statement

The present study was conducted in accordance with the guidelines of the Helsinki Declaration of the World Medical Association. The study was approved by the national ethics committees (Hungarian Scientific and Research Ethics Committee of the Medical Research Council, ETT TUKEB 7214-1/2016/EKU [0109/16], ÁNTSZ IF-77-3/2016, SE IKEB 241/2016). The need for individual informed consent for this retrospective study was waived. After the collection of clinical information, patient identifiers were removed, and subsequently, patients could not be directly or indirectly identified.

### Patients

Our retrospective study included 66 pathologically confirmed rare lung tumors including 26 mucoepidermoid carcinomas (MECs), 27 adenoid cystic carcinomas (ACCs), and 13 tracheobronchial papillomas (TBPs). Patients were diagnosed and treated in the National Koranyi Institute of Pulmonology in Budapest between 1987 and 2019, therefore, all tumor samples were re-examined. The ACC and MEC diagnoses were confirmed with the following IHC markers: pancytokeratin, actin, S100, and p63.

The clinicopathological characteristics of patients including age, gender, smoking history, histology, stage and the type of biopsy material are summarized in Table 1.

**TABLE 1:** Major characteristics of 66 patients diagnosed with and treated for mucoepidermoid carcinoma, adenoid cystic carcinoma, and tracheobronchial papilloma between 1987 and 2019.

Variables		MEC	ACC	TBP
Number of cases		26	27	13
Gender	Male	16 (62%)	9 (33%)	11 (85%)
	Female	10 (38%)	18 (67%)	2 (15%)
Mean age, range (years)		43.4 (24–67)	54.4 (32–83)	54.2 (21–80)
Smoking history, never/ex/current/nd		14/1/6/5	11/6/5/5	3/1/4/5
Bronchoscopic excision/Surgical material		7/19	18/9	12/1
Stage	IA/IB	9/8	0/7	
	IIA/IIB	0/3	0/1	
	IIIA/IIIB	2/0	16/0	
	IV/nd	1/3	0/3	

MEC, mucoepidermoid carcinoma; ACC, adenoid cystic carcinoma; TBP, tracheobronchial papilloma; nd, no data.

### Immunohistochemistry

In the case of surgical specimens immunohistochemical IHC staining was performed on tissue microarrays (TMAs) (5 × 6; diameter, 2 mm) with double or triple cores per patient, prepared from selected areas of formalin fixed paraffin embedded (FFPE) tissue samples (TMA Master; 3DHistech, Budapest, Hungary). In the case of bronchoscopic excisions 3-µm-thick sections were prepared for IHC without TMA construction.

Ventana SP142 was first used as a primary antibody for PD-L1 (clone SP142, dilution 1:100; Spring Bioscience, Ventana; Oro Valley, AZ). However, we did not detect any immunoreaction either on tumor cells (TC) or on immune cells (IC), therefore, we subsequently applied PharmDx 22C3 antibody. For PD-1 staining Abcam 52587 antibody was used. The mouse monoclonal PD-L1 and PD-1 antibodies (PharmDx, clone 22C3, ready to use, and Abcam, clone NAT105, dilution 1:50, respectively) were used for IHC in line with standardized protocols. After deparaffinization and rehydration, the sections were incubated in a 3% H_2_O_2_ solution for 10 min in order to reduce the nonspecific background staining. It was heated afterwards for 40 min in a 10 mM citrate buffer (pH 6.0) at 98°C. Slides were then incubated for 5 min at room temperature with Ultra V Block (UltraVision LP detection system, Lab Vision Corporation, Thermo Fisher Scientific Inc., Pittsburgh, MA, United States), followed by PD-L1 and PD-1 antibody incubation for overnight at 4°C. Immunoreaction was detected using the UltraVision LP Detection System following the manufacturer’s recommendations (Lab Vision Corporation). The antibody visualization was performed with 3–3′-diaminobenzidine (DAB) and counterstained with hematoxylin. As a positive control, placental tissue was used.

All sections were examined and evaluated by an experienced pathologist at ×400 magnification. The amount of positive TC and IC was determined by a semi-quantitative method as the percentage of positive cells. For TC 1%, 5%, and 50%, while for IC 1%, 5%, and 10% cut off levels were recorded, which are the most commonly used thresholds and wich we also used in our previous studies [10–15].

### Statistical analysis

All statistical analyses were performed using SPSS Statistics 25.0 package (SPSS Inc., Chicago, IL, United States). Dichotomization concerning age was performed by using one of the widely used threshold values in cancer studies (55 years) [16]. Due to the low number of cases, categorical parameters, such as age (≤55 years vs. >55 years), gender (male vs. female) and smoking history (never smoker vs. ex-smoker vs. smoker) were analyzed by Fisher’s exact test. Different PD-L1/PD-1 subgroups (<1% vs. ≥1%) were also compared using Fisher’s exact test. All reported *p* values were two-sided, and a level of 0.05 or lower was considered statistically significant.

## Results

### PD-L1 expression

Since the first immunostaining for PD-L1 with Ventana SP142 antibody did not yield positive immunoreaction on any of the slides, we also used PharmDx 22C3 antibody.

**FIGURE 1:**
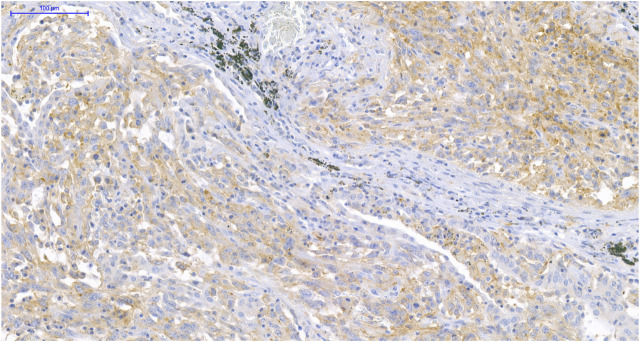
PD-L1 immunohistochemistry (IHC) of MEC. IHC shows >50% PD-L1 positive TCs and >10% PD-L1 positive ICs in MEC (×200).

PD-L1 expression on TCs was observed in two cases of MEC (7.7%), one case of ACC (3.7%), and was absent in TBP samples, while PD-L1 expression on ICs could be demonstrated in nine cases of MEC (34.6%), four cases of ACC (14.8%), and was absent in TBPs. Although these ratios indicate a higher positivity rate in MEC compared to ACC, the difference was not significant (*p* = 0.119). All PD-L1 TC positive tumors were also PD-L1 IC positive. Expression level higher than 5% of PD-L1 TC and IC was observed only in one ACC and in two MEC patients. Among them only one female smoker patient with MEC presented with strong PD-L1 expression both on TCs (≥50%) and ICs (≥10%) (Figure 1); a male patient with MEC had 5%–49% PD-L1 TC level, and another male patient with ACC had 5%–9% PD-L1 IC level.

### PD-1 expression

PD-1 expression on ICs was observed in five cases of MEC (19.2%), four cases of ACC (14.8%), and two cases of TBP (15.4%). A higher than 5% expression level of PD-1 on ICs was observed only in one MEC case.

Since we only found very few samples with higher than 5% PD-L1/PD-1 expression on TCs and ICs, we used dichotomized categories (i.e., positive [≥1%] vs. negative [<1%]) for statistical analysis.

### Correlation between PD-1/PD-L1 expression and clinicopathological findings

We examined the potential correlation between clinicopathological findings and the different PD-1/PD-L1 expression profiles of the tumors. In MEC cases, PD-L1 expression levels of ≥1% on TCs and ICs were significantly more common in patients aged 55 years or older, than in younger patients (*p* = 0.046, and *p* = 0.01, respectively). Apart from these observations, no other significant correlation was found between PD-L1/PD-1 expression levels and patient characteristics, such as age, gender or smoking history. These results are summarized in Tables 2–4.

**TABLE 2:** Association between PD-L1 expression on TCs and clinicopathological factors.

Clinicopathological characteristics		MEC (*n* = 26)			ACC (*n* = 27)			TBP (*n* = 13)		
		PD-L1 TC <1%	PD-L1 TC ≥ 1%	*p*-value^a^	PD-L1 TC <1%	PD-L1 TC ≥ 1%	*p*-value^a^	PD-L1 TC <1%	PD-L1 TC ≥ 1%	*p*-value^a^
Age (years)	≤55	20	0	**0.046** ^b^	11	1	0.444^b^	7	0	0.999^b^
	>55	4	2		15	0		6	0	
Gender	Male	15	1	0.999^b^	8	1	0.333^b^	11	0	0.999^b^
	Female	9	1		18	0		2	0	
Smoking history	Never smoker	13	1	0.604^b^	10	1	0.681^b^	3	0	0.999^b^
	Ex-smoker	1	0		6	0		1	0	
	Smoker	5	1		5	0		4	0	
	nd	5	0		5	0		5	0	

^a^ *p* values refer to PD-L1 negative versus PD-L1 positive subgroups.

^b^ Fisher’s exact test was used between categorical variables; MEC, mucoepidermoid carcinoma; ACC, adenoid cystic carcinoma; TBP, tracheobronchial papilloma; nd, no data; TC, tumor cell; PD-L1, programmed death ligand-1.

Bold represents statistically significant *p* value.

**TABLE 3:** Association between PD-L1 expression on ICs and clinicopathological factors.

Clinicopathological characteristics		MEC (*n* = 26)			ACC (*n* = 27)			TBP (*n* = 13)		
		PD-L1 IC <1%	PD-L1 IC ≥ 1%	*p*-value^a^	PD-L1 IC <1%	PD-L1 IC ≥ 1%	*p*-value^a^	PD-L1 IC <1%	PD-L1 IC ≥ 1%	*p*-value^a^
Age (years)	≤55	16	4	**0.01** ^b^	11	1	0.999^b^	7	0	0.999^b^
	>55	1	5		12	3		6	0	
Gender	Male	12	4	0.667^b^	7	2	0.581^b^	11	0	0.999^b^
	Female	5	5		16	2		2	0	
Smoking history	Never smoker	9	5	0.613^b^	9	2	0.582^b^	3	0	0.999^b^
	Ex-smoker	1	0		5	1		1	0	
	Smoker	3	3		4	1		4	0	
	nd	4	1		5	0		5	0	

^a^ *p* values refer to PD-L1 negative versus PD-L1 positive subgroups.

^b^ Fisher’s exact test was used between categorical variables; MEC, mucoepidermoid carcinoma; ACC, adenoid cystic carcinoma; TBP, tracheobronchial papilloma; IC, immune cell; nd, no data; PD-L1, programmed death ligand-1.

Bold represents statistically significant *p* value.

**TABLE 4:** Association between PD-1 expression on ICs and clinicopathological factors.

Clinicopathological characteristics		MEC (*n* = 26)			ACC (*n* = 27)			TBP (*n* = 13)		
		PD-1 IC <1%	PD-1 IC ≥ 1%	*p*-value^a^	PD-1 IC <1%	PD-1 IC ≥ 1%	*p*-value^a^	PD-1 IC <1%	PD-1 IC ≥ 1%	*p*-value^a^
Age (years)	≤55	17	3	0.558^b^	11	1	0.999^b^	6	1	0.999^b^
	>55	4	2		12	3		5	1	
Gender	Male	13	3	0.999^b^	7	2	0.582^b^	9	2	0.999^b^
	Female	8	2		16	2		2	0	
Smoking history	Never smoker	11	3	0.613^b^	9	2	0.582^b^	3	0	0.218^b^
	Ex-smoker	1	0		5	1		1	0	
	Smoker	4	2		4	1		4	0	
	nd	5	0		5	0		3	2	

^a^ *p* values refer to PD-1 negative versus PD-1 positive subgroups.

^b^ Fisher’s exact test was used between categorical variables; MEC, mucoepidermoid carcinoma; ACC, adenoid cystic carcinoma; TBP, tracheobronchial papilloma; IC, immune cell; nd, no data; PD-1, programmed death-1.

The association between tumor histology and PD-L1 expression on TCs and ICs, and PD-1 expression on ICs was also examined. A PD-L1 expression level of ≥1% on ICs were significantly more common in MEC, than in TBP (*p* < 0.001) (Table 5). No other significant correlations were observed.

**TABLE 5:** Association between the tumor histology and PD-L1 expression on TCs/ICs and PD-1 expression on ICs.

PD-L1 TC	MEC (%)	ACC (%)	TBP (%)	*p*-value^a^	PD-L1 IC	MEC (%)	ACC (%)	TBP (%)	*p*-value^a^	PD-1 IC	MEC (%)	ACC (%)	TBP (%)	*p*-value^a^
<1%	24 (92.3%)	26 (96.3%)	13 (100%)	0.192^b^	<1%	17 (65.4%)	23 (85.2%)	13 (100%)	**<0.001** ^b^ ^,^ ^c^	<1%	21 (80.8%)	23 (85.2%)	11 (84.6%)	0.082^b^
≥1%	2 (7.7%)	1 (3.7%)	0 (0%)		≥1%	9 (34.6%)	4 (14.8%)	0 (0%)		≥1%	5 (19.2%)	4 (14.8%)	2 (15.4%)	

^a^ *p* values refer to PD-L1/PD-1 negative versus PD-L1/PD-1 positive subgroups.

^b^ Fisher’s exact test was used between categorical variables.

^c^ A PD-L1 expression level of ≥1% on ICs were significantly more common in MEC, than in TBP; MEC, mucoepidermoid carcinoma; ACC, adenoid cystic carcinoma; TBP, tracheobronchial papilloma; TC, tumor cell; IC, immune cell; nd, no data; PD-1, programmed death-1; PD-L1, programmed death ligand-1.

Bold represents statistically significant *p* value.

Since the age of FFPE blocks can influence the IHC reactions, we compared older and newer tissue materials, however, we found no statistically significant difference in IHC positivity rates between pre- and post-2006 samples.

## Discussion

The role of immunotherapy in the treatment of MEC and ACC has not been extensively studied, and the majority of available publications focus on head and neck salivary gland tumors. In a recent case report successful treatment with low-dose radiotherapy combined with immunotherapy was described in a patient with metastatic submandibular salivary gland ACC, however, no such data have been reported in lung ACC so far [17]. Farhat et al. reported a case of advanced high-grade MEC of the parotid salivary gland after pembrolizumab treatment as a first line therapy [18]. The tumor was downstaged as a result of the immunotherapy, allowing for surgical excision. Recently, two cases of metastatic high-grade MECs presented as left neck mass with prolonged response to pembrolizumab have been published [19]. In 2022, a comprehensive overview summarized novel therapeutic approaches including targeted therapy and immunotherapy in different subtypes of salivary gland cancer [20]. However, this publication only focused on head and neck tumors. In a very recent publication, Hu et al. provided a thorough overview of the main diagnostic and therapeutic implications of pulmonary MEC including targeted therapy, chemotherapy, and immunotherapy [21]. They confirm that the immunotherapy approach for this type of tumor is limited so far and needs to be explored in-depth. They reported only one publication dealing with PD-L1/PD-1 expression in lung MEC, however, this article was published in Fujian Medical Journal, and is not available on PubMed [22].

The potential predictive value of PD-L1/PD-1 expression for the efficacy of immunotherapy in salivary gland tumors is not yet understood, and the majority of publications related to ACC focus on PD-L1 and PD-1 expression in head and neck tumors [23]. Zheng et al. compared the PD-L1 expression in tracheobronchial ACC and SCC, and found no expression in the studied 16 ACC samples, whereas 80% of SCCs were PD-L1 positive [9]. Their methodology was similar to our current study in terms of the applied clone (PD-L1 22C3 clone) and the threshold for PD-L1 positivity (expression ≥1%). In our present work PD-L1 was positive in 1/27 ACC tumors on TCs, and in 4/27 tumors on ICs. In one of our MEC samples, a strong PD-L1 immunopositivity of >50% could be observed on TCs.

Witte et al. investigated PD-L1 expression on ICs in 94 head and neck salivary gland carcinomas including 41 ACCs, 21 MECs, 16 acinic cell carcinomas, 12 adenocarcinomas not otherwise specified, 2 epithelial-myoepithelial carcinomas, one salivary duct carcinoma, and one carcinoma ex pleomorphic adenoma. Using SP263 clone for PD-L1 IHC, they identified significantly elevated IC scores in adenocarcinomas not otherwise specified compared to ACC and MEC [24]. In our study, PD-L1 positivity on ICs was more common in MEC than in ACC (34.6% vs. 14.8%), although the difference was not statistically significant. Higher than 5% expression levels were observed in three MEC samples, and in one case it was even >10%.

In salivary gland tumors, data regarding PD-1 expression are even more scarce, than on PD-L1 expression. In our study, PD-1 expression on ICs was found in 19.2% of MEC, 14.8% of ACC, and 15.4% of TBP samples. This is lower than what Xu et al. observed in salivary duct carcinoma, where PD-1 positivity on ICs was noted in 66% of cases, but higher than in the study by Mosconi et al. which found scarce PD-1 positivity in 36 salivary gland ACCs [25, 26].

TBPs are often treated with bronchoscopic excision, and radical removal of the tumor usually results in complete recovery. In recurrent cases and/or in tracheobronchial papillomatosis, there is a risk of malignant transformation to SCC, and bronchoscopic tumor resection is no longer feasible in extensive disease. Therefore, it is important to determine the potential role of immunotherapy in irresectable cases. Currently, PD-L1 expression on TCs and ICs is the most widely used predictive biomarker for immunotherapy in head and neck SCC. Nevertheless, patients with PD-L1 negative tumors may still respond to treatment. In our present study, we found no PD-L1 expression on TC-s or ICs in TBP tumor samples, and only 2 TBP cases (15.4%) showed weak PD-1 positivity on ICs.

Several antibodies are used to examine PD-L1 protein expression. At the beginning of the 2010s, SP142 proved to be the most suitable antibody. A few years later a comparative study was carried out, in which the goal was to analyze four commercially available antibodies that had already appeared in clinical practice. During this, it was determined that in lung cancer, Ventana’s SP142 antibody is less sensitive than the other three antibodies (Ventana SP263, Dako 22C3, Dako 28–8) [27]. The results of our present study also confirm this observation, as no IHC positive samples were found when SP142 antibody was used initially, and highlight the importance of using anti-PD-L1 antibody with proper sensitivity in order to avoid false negative IHC results.

In lung cancer, indications for immunotherapy are constantly expanding. Previously, it was mainly used in advanced-stage NSCLC as second- or third line treatment, however, nowadays the range of indications extends to the first line treatment, usually combined with chemotherapy [2]. Furthermore, the role of immunotherapy in the neoadjuvant and adjuvant settings is also being investigated [28]. It has been clearly demonstrated that the most favorable therapeutic response can be expected in case of >50% PD-L1 expression, however, patients with PD-L1 negative tumors may also respond to immunotherapy [29–31]. Furthermore, many clinical trials use the PD-L1 cut-off value of 1% as an inclusion criterion. In our work 34.6% of MEC and 14.8% of ACC cases belonged to the ≥1% PD-L1 IC group, and higher than 5% expression levels on TC and/or IC were detected in one ACC and two MEC samples.

Lung cancer subtypes previously considered homogeneous are increasingly being recognized as heterogeneous, which has important therapeutic implications. In lung adenocarcinoma, the determination of the molecular subtype and PD-L1 expression level is now essential for treatment decision-making. Although certain molecular subtypes comprise only few patients, such as Her2 (3%), MET (3%), RET (1%), and ROS1 (1%), targeted therapy may offer significant benefits for them [32]. Similarly, small cell lung cancer is no longer considered a homogenous group, since four molecular subgroups have been discovered, which also have major therapeutic implications [33, 34]. The different PD-L1 and PD-1 expression levels in MEC and ACC tumors found in our study raise the possibility of heterogeneity in these groups, too, which seems to be supported by recent molecular genetic studies in salivary gland ACC [35].

Notwithstanding, our results might suffer from the limitations of having a relatively small number of cases. Undoubtedly, a significantly larger cohort would give more reliable insights into the expression of the described biomarkers in MEC, ACC and TBP, however, in rare tumors, usually it takes many years to collect the number of cases suitable for such examinations. Other limitation might be the age of FFPE tissue samples, which may affect IHC reactions. In order to investigate this, we compared the IHC results of early and late tissue samples and found no significant difference.

Based on PubMed publications, our study is the first to report comprehensive data on PD-L1/PD-1 expressions in primary pulmonary MEC and TBP, and confirms the results of the very few published studies in lung ACC. In these rare pulmonary tumors, there is unmet need to develop more efficient therapeutic modalities, especially for patients with advanced-stage disease. Our results demonstrate that primary lung salivary gland tumors are heterogeneous not only in terms of the MEC/ACC subgroups, but also within these histologic subtypes. A more comprehensive exploration of this observation in a larger cohort may contribute to personalized therapy in rare lung cancers. If ≥50% PD-L1 TC in MEC is confirmed even if only in a low proportion of patients, MEC with high PD-L1 expression might be regarded as a separate subgroup with distinct treatment option, such as immune checkpoint inhibition therapy.

## Data Availability

The original contributions presented in the study are included in the article/supplementary material, further inquiries can be directed to the corresponding author.

## Abbreviations

ACC, adenoid cystic carcinoma; ADC, adenocarcinoma; IC, immune cell; IHC, immunohistochemistry; MEC, mucoepidermoid carcinoma; NSCLC, non-small cell lung cancer; PD-1, programmed cell death-1; PD-L1, programmed cell death ligand-1; SCC, squamous cell carcinoma; TBP, tracheobronchial papilloma; TC, tumor cell.
